# Overexpression of SYNGAP1 suppresses the proliferation of rectal adenocarcinoma via Wnt/β-Catenin signaling pathway

**DOI:** 10.1007/s12672-024-00997-z

**Published:** 2024-04-29

**Authors:** Yun Xiao, Ying Zhu, Jiaojiao Chen, Mei Wu, Lan Wang, Li Su, Fei Feng, Yanli Hou

**Affiliations:** Department of Oncology and Hematology, Chongqing Hospital of Traditional Chinese Medicine, Chongqing, China

**Keywords:** Ras GTPase-activating proteins, Rectal adenocarcinoma, SYNGAP1, Wnt/β-Catenin signaling pathway, Prognosis, Methylation

## Abstract

Rectal adenocarcinoma (READ) is a common malignant tumor of the digestive tract. Growing studies have confirmed Ras GTPase-activating proteins are involved in the progression of several tumors. This study aimed to explore the expression and function of Ras GTPase-activating proteins in READ. In this study, we analyzed RNA sequencing data from 165 patients with READ and 789 normal tissue samples, identifying 5603 differentially expressed genes (DEGs), including 2937 upregulated genes and 2666 downregulated genes. Moreover, we also identified two dysregulated genes, RASA4 and SYNGAP1, among six Ras GTPase-activating proteins. High NF1 expression was associated with longer overall survival, while high SYNGAP1 expression showed a trend towards extended overall survival. Further analysis revealed the mutation frequency and copy number variations of Ras GTPase-activating proteins in various cancer samples. Additionally, DNA methylation analysis demonstrated a negative correlation between DNA methylation of Ras GTPase-activating proteins and their expression. Moreover, among Ras GTPase-activating proteins, we focused on SYNGAP1, and experimental validation confirmed that the overexpression of SYNGAP1 in READ significantly suppressed READ cell proliferation and increased apoptosis via regulating the Wnt/β-Catenin signaling pathway. These findings underscored the potential significance of SYNGAP1 in READ and provide new insights for further research and treatment.

**Supplementary Information:**

The online version contains supplementary material available at 10.1007/s12672-024-00997-z.

## Introduction

With a total of 140,250 new cases in 2018, accounting for 8.1% of all new cancer cases, rectal adenocarcinoma (READ) is the main cause of death resulting from cancer [1]. Due to the high incidence and fatality rates, READ is considered to be a type of that plays a significant impact. READ typically occurs more frequently in middle-aged and elderly individuals, especially those aged 50 and older. Age is an important risk factor for rectal adenocarcinoma [2]. Research has found that men are more likely to develop rectal adenocarcinoma compared to women. This gender difference may be associated with male lifestyle, hormonal levels, and other biological factors [3–5]. Individuals with a family history of READ have a higher risk of developing the disease. If there is a history of rectal adenocarcinoma or other READ in the family, the individual's risk of developing the disease may be increased. Some genetic mutations are also associated with rectal adenocarcinoma, such as and Familial Adenomatous Polyposis (FAP) [6]. The overall survival rates for patients who have been diagnosed with advanced rectal adenocarcinoma remain low despite major breakthroughs in screening as well as surgery combined with treatment [7–9]. Thus, it is of the utmost importance to investigate the molecular mechanisms of READ progression and to search for more precise tumor prognostic indicators that may reliably predict the survival of patients who have READ.

Ras proteins (Rat Sarcoma) are a family of small GTPase proteins, including subtypes such as H-Ras, K-Ras, and N-Ras. They play a crucial role in cell signaling, particularly in regulating cell proliferation, growth, and differentiation [10]. Mutations or abnormal activation of Ras proteins are closely associated with the development and progression of various cancers [11]. In some cancers, Ras proteins are permanently locked in their activated state, leading to excessive cell proliferation and the formation of cancer [12, 13]. Therefore, Ras proteins are considered as potential targets for cancer therapy. Ras GTPase-activating proteins, commonly abbreviated as RasGAPs, are a class of proteins that play a crucial role in cell signaling processes. Their primary function is to regulate the activity of Ras proteins, particularly one of the Ras family proteins, such as H-Ras, K-Ras, and N-Ras [14]. Ras proteins are GTPases, capable of switching between an active state (GTP-bound) and an inactive state (GDP-bound), thus influencing downstream signaling pathways. RasGAPs facilitate this process by accelerating the GTP hydrolysis reaction of Ras proteins, assisting Ras in transitioning from the GTP-bound state to the GDP-bound state. The process is considered a key negative feedback mechanism within the Ras signaling pathway, contributing to the maintenance of cellular signal transduction equilibrium [15, 16]. In recent years, several studies have reported that Ras GTPase-activating proteins are involved in the progression of several tumors [17, 18]. In some tumors, the expression levels of Ras GTPase-activating proteins (Ras GAPs) may be abnormal, either through overexpression or underexpression. Overexpression of Ras GAPs may lead to excessive inhibition of the Ras signaling pathway, thereby affecting cell proliferation and survival, while underexpression may result in excessive activation of the Ras signaling pathway, promoting tumor development. However, the expression and function of Ras GTPase-activating proteins in READ were rarely reported.

In this study, we firstly carried out Bioinformatics analysis using TCGA datasets to explore the expression and prognostic value of Ras GTPase-activating proteins in READ. Then, we further explored the SNV, CNV landscape and Methylation of Ras GTPase-activating proteins in pan-cancers. Among Ras GTPase-activating proteins, our attention focused on SYNGAP1 and performed functional experiments to explore its expression and function in READ progression. These research findings are of significant importance as they provide valuable information for understanding the onset and progression of cancer, while also offering new leads for potential clinical applications and treatment strategies.

## Materials and methods

### Cell culture and cell transfection

The use of Transfection and Cell Culture Leibovitz's 15 (L-15) culture media (ATCC) supplemented with 10% fetal bovine serum (FBS; ATCC) was used to cultivate the human rectal mucosa epithelial cell line (PriCells) and two types of human READ cell lines (SW837 and SW1463; ATCC). Logarithmic-phase cells were collected for further study. Genechem (Shanghai, China) provided both the pcDNA3.1 vector targeting SYNGAP1 and the empty vector. These plasmids were used in a Lipofectamine 3000 (Invitrogen) transfection to introduce them into the cells.

### Cell proliferation assay

In the CCK-8 cell experiment, the first step was to culture the SW837 and SW1463 cell lines to ensure their viability under appropriate culture conditions. Subsequently, both cell types are harvested and counted to ensure that the same number of cells is used in the experiment. Next, the cell suspensions are distributed into a 96-well plate, with an equal number of cells in each well. CCK-8 cell assay reagent is added to each well and diluted in the culture medium. The plate was then incubated at 37 degrees Celsius for 1 to 4 h to allow the cells to react with the CCK-8 reagent. After the reaction is complete, the absorbance of each well is measured using a microplate reader at a wavelength of 450 nm.

In this clonogenic formation experiment, initially, 1 × 10^3^ cells were seeded in each well of a 6-well culture plate, followed by incubation at 37 °C for one week. The incubation period allowed sufficient time for the cells to grow and form clones. Subsequently, the cells were washed twice with PBS to remove unattached cells and residual culture medium. Afterward, the cells were fixed using a 4% formaldehyde solution for 15 min to stabilize their cellular structures and preserve their morphology. Next, the cells were stained with GIMSA, a commonly used staining method for observing cell morphology and structure under a microscope. Staining typically lasted for 10–30 min, with the specific duration adjusted as needed for the experiment. Finally, the colonies with a diameter greater than or equal to 100 µm were counted in triplicate assays.

### RNA isolation and qRT-PCR

The READ cells are cultured under appropriate conditions until reaching the desired density. We employ aseptic techniques to transfer the cells from the culture dish into centrifuge tubes or other suitable collection containers. The TRIzol reagent (Invitrogen, Carlsbad, CA, USA) was used to isolate the RNA. The RNA precipitate is washed with ethanol to remove residual salts and TRIzol residues. The washing process is repeated multiple times to ensure thorough cleansing. SYNGAP1 levels were determined using the StepOneTM Real-Time PCR System (Applied Biosystems, Carlsbad, CA, USA) and SYBR Green (Applied Biosystems). Endogenous control for SYNGAP1 was determined using GAPDH. SYNGAP1 expression levels in READ cells were compared using a 2-Ct method. The primers for SYNGAP1 were as follows: sense, CCTTCAGAGATGTACGGGGAC, and antisense, GTTCCAACCAGGACGATCATAC. GAPDH: sense, GGAGCGAGATCCCTCCAAAAT, and antisense, GGCTGTTGTCATACTTCTCATGG.

### TUNEL assay

After 30 min in 4% paraformaldehyde, the cells were fixed in 70% cold alcohol. At room temperature, the cells were treated with PBS containing 0.3% Triton X-100 for 5 min. Cells were treated with a TUNEL solution, which typically contains a labeled nucleotide (often dUTP) and the enzyme terminal deoxynucleotidyl transferase (TdT). This enzyme adds the labeled nucleotide to the 3′-OH ends of fragmented DNA. The reaction was carried out for 60 min at 37 °C in the dark. This step specifically labels the DNA fragments that are characteristic of apoptotic cells. After the TUNEL reaction, the cells were likely washed to remove any unincorporated TUNEL reagents. The cells were then mounted with an anti-fluorescence quenching agent, which helps reduce background fluorescence, and observed under a fluorescence microscope. The TUNEL-labeled DNA fragments will emit fluorescence, and this can be visualized and quantified. Finally, cell nuclei were stained with Hoechst.

### Western blot

The cells were lysed using the cell lysis solution, and the resulting proteins were harvested. After adding 5 loading buffer, the mixture was brought to a boil for 10 min. Under a 300 mA constant current for 60–90 min, polyvinylidene fluoride (PVDF) membranes were transferred after SDS-PAGE. Primary anti-bodies for -catenin (1:8000, Abcam), c-Myc (1:1000, Cell Signaling Technology), and Cyclin D1 (1:1000, Cell Signaling Technology) were added after the PVDF membrane had been sealed with 5% skimmed milk powder for 2 h. Secondary antibodies conjugated with horse radish peroxidase (HRP) (1:15,000, Jackson Immuno Research Inc) were added to the cells after another overnight incubation at 4 °C. The grayscale analysis was completed with the addition of a freshly prepared ECL luminescent solution. Before antibody hybridization, the membrane was cropped to ensure accurate detection of the regions of interest.

### Data acquisition

The Cancer Genome Atlas (TCGA) Genomic Data Commons Data Portal (https://portal.gdc.cancer.gov/) was accessed to retrieve the RNA-seq mRNA expression profiles and clinical data of the TCGA-READ cohorts. In addition, normal samples were downloaded from GTEx datasets (n = 779). Patients were only considered if they had pathologically verified READ and full information about transcriptomics OS. In the end, we used 165 READ instances from TCGA databases for our research. TCGA barcodes were used to first filter out paired-normal samples from TCGA-READ cohorts. Next, we converted FPKM data to TPM. The average expression value of replicate samples from individual patients was determined.

### Identification of differentially expressed genes (DEGs) in READ

The DEGs were computed with the help of the Limma package in R, version 3.36.2. Only differentially expressed genes (DEGs) with an absolute log2 fold change (FC) > 2 and an adjusted P value 0.05 were included in the study.

### Functional and Pathway Enrichment Analyses

ClusterProfiler is an R package used for functional enrichment analysis and visualization of gene sets in the field of bioinformatics [19]. This package is primarily employed to interpret high-throughput biological data, such as gene expression data, proteomics data, metabolomics data, etc., to aid researchers in understanding the biological functions and associations of different gene sets. The R package clusterProfiler (V3.14.3) was used to construct GO and KEGG pathways, which clarified the role of genes in the central modules. ClusterProfiler leverages the GO.db (V3.14) and KEGG.db (V3.2.4) databases from Bioconductor to assign GO and KEGG names to genes, respectively. Each DEG is assigned to specific KEGG pathways based on its known or predicted functions. This step involves mapping DEGs to their corresponding pathways within the KEGG database. The KEGG annotation of the signaling transduction pathway associated with DEGs complements the function annotation of these genes. The threshold p-value chosen was 0.05.

### CNV and methylation analysis

The Gene Set Cancer Analysis (GSCA) is a bioinformatics tool and method designed for the comprehensive analysis of cancer-related gene sets. It is used to explore the roles and associations of specific gene sets in the context of cancer biology. GSCA integrates various data sources, including gene expression data, clinical information, and biological knowledge, to provide insights into the relationships between gene sets and cancer subtypes, progression, or patient outcomes. CNVs and methylation of Ras GTPase-activating proteins were analyzed using the "mutation" module of the GSCA database.

### Statistical analysis

All bioinformatic data was filtered by removing missing and duplicate information, and statistical and graphical work was performed in R (version 3.6.3). In order to compare the levels of Ras GTPase-activating proteins in cancerous and noncancerous tissues, we utilized the Wilcoxon rank-sum test and the “ggplot2” package (version 3.3.3) to generate graphs of our findings. For the comparison of clinical data between the two groups, a t-test for continuous variables was applied. Notably, our statistical analyses encompassed variance analysis and t-tests. The log-rank test was run after Kaplan–Meier survival curves were drawn. We used a t-test for continuous variables to compare clinical data from the two groups. Statistical analysis and visualization were performed using GraphPad Prism 8, and a p value of less than 0.05 was considered significant.

## Results

### Identification of DEGs in READ from TCGA datasets and GTEx datasets

**Fig. 1:**
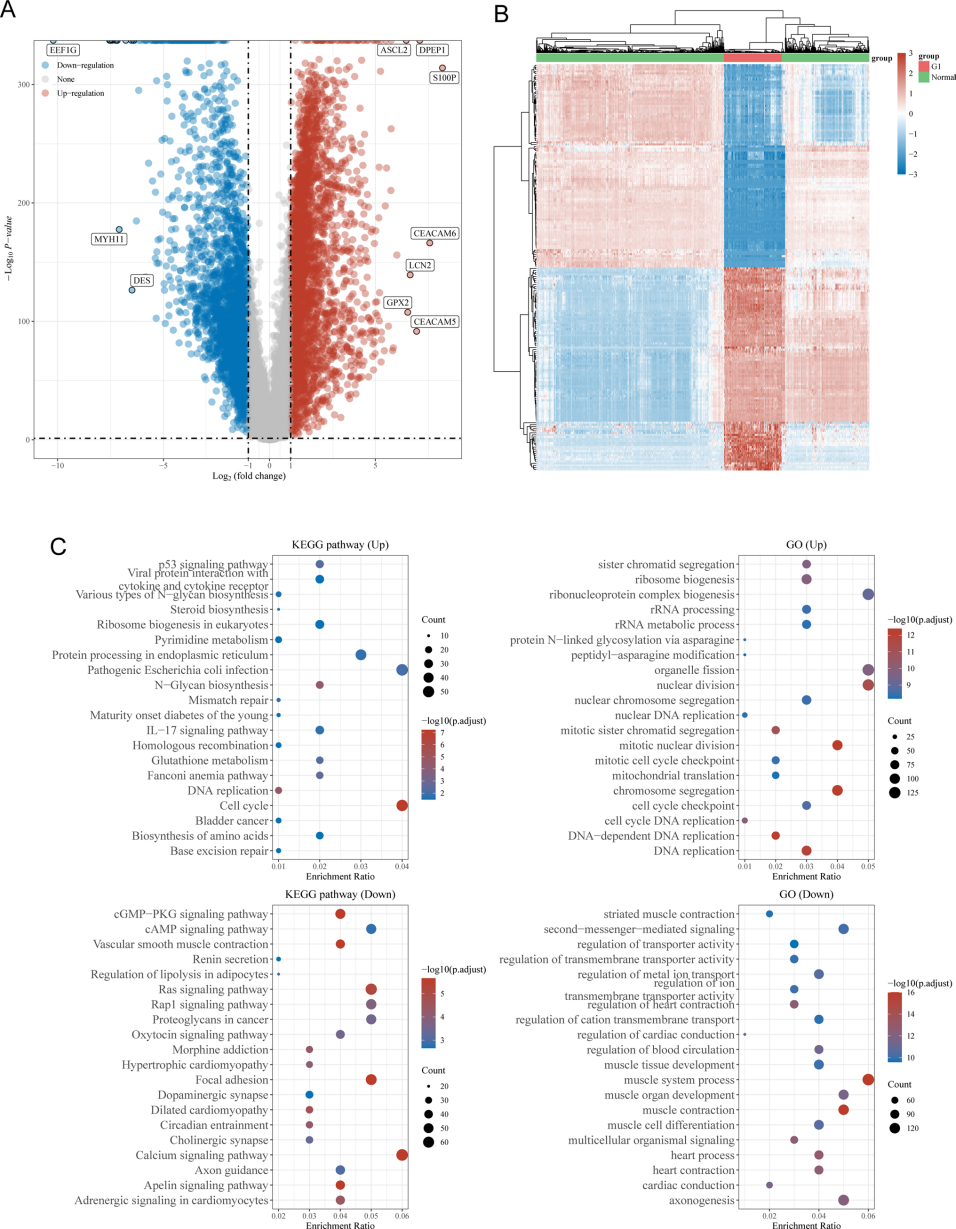
Identification of differentially expressed genes (DEGs) in READ and functional enrichment analysis. **A** Volcano map of DEGs. **B** Visualization of TCGA-derived DEGs as a heatmap. **C** KEGG and GO analyses of DEGs that were up- or down-regulated

RNA-seq data from the TCGA database and the GTEx datasets were used for the DEGs study, and they included a total of 789 normal tissue samples and 165 READ. In this study, we screened out a total of 5603 DEGs, including 2937 up-regulated and 2666 down-regulated genes, based on the screening criteria of FDR < 0.05 and |log2 FC|> 2(Fig. 1A and B).

### GO functional and KEGG pathway enrichment analysis of DEGs

To explore the potential function of DEGs, we performed GO and KEGG assays. We observed that 2937 up-regulated genes were mainly enriched in p53 signaling pathway, Viral protein interaction with cytokine and cytokine receptor, Various types of N-glycan biosynthesis and Steroid biosynthesis, and 2666 down-regulated genes were mainly enriched in cGMP-PKG signaling pathway, cAMP signaling pathway, Vascular smooth muscle contraction and Renin secretion. In addition, the results of GO analysis revealed that 2937 up-regulated genes were mainly related to sister chromatid segregation, ribosome biogenesis, ribonucleoprotein complex biogenesis and rRNA processing, and the 2666 down-regulated genes were mainly related to striated muscle contraction, second-messenger-mediated signaling, regulation of transporter activity and regulation of transmembrane transporter activity(Fig. 1C).

### The expression pattern of Ras GTPase-activating proteins in READ

**Fig. 2:**
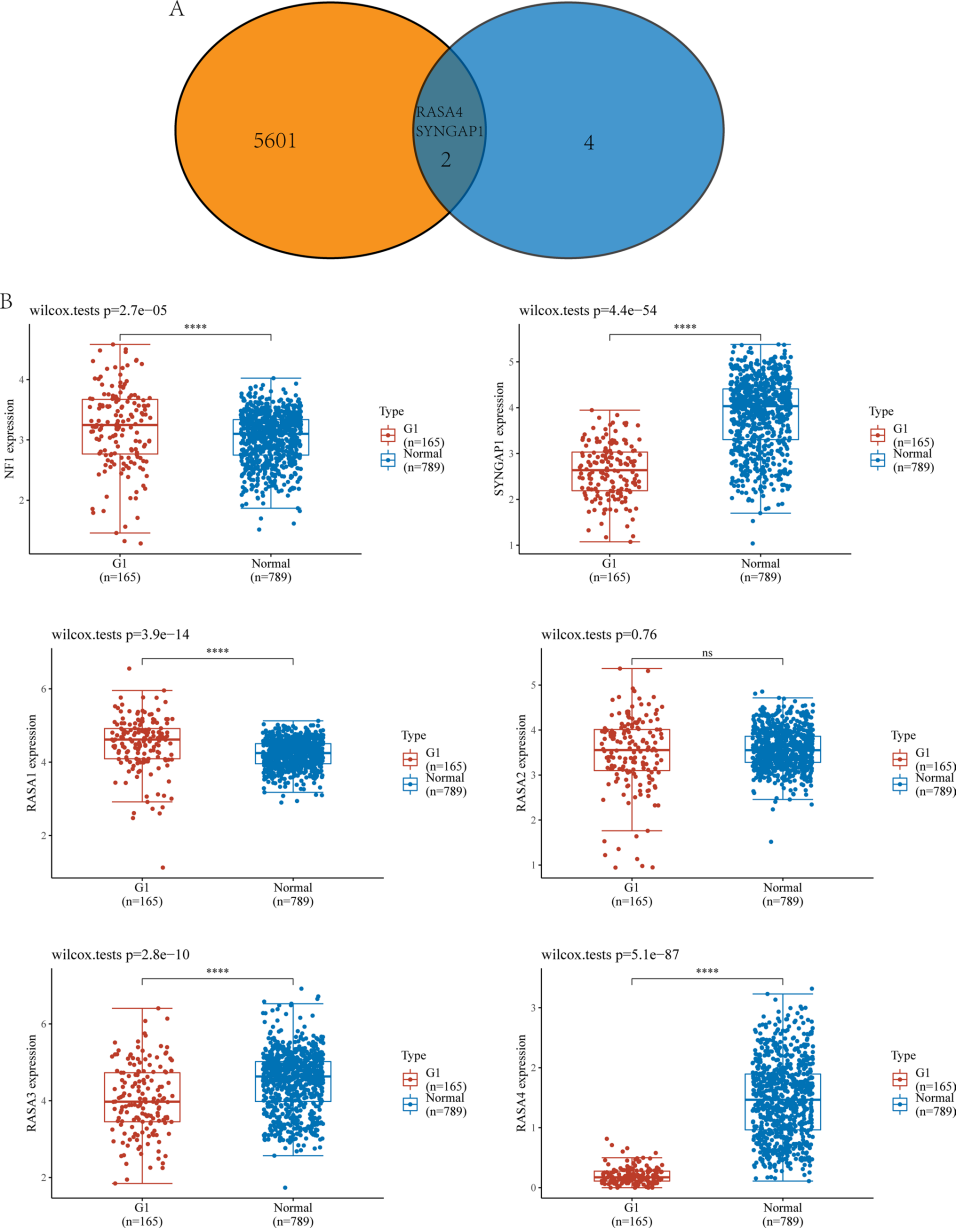
The expressing pattern of Ras GTPase-activating proteins in READ patients. **A** Venn Diagram showed RASA4 and SYNGAP1 exhibited a dysregulated level in READ samples compared with normal samples. **B** The expressing pattern of Ras GTPase-activating proteins in READ samples and normal samples from TCGA datasets

**Fig. 3:**
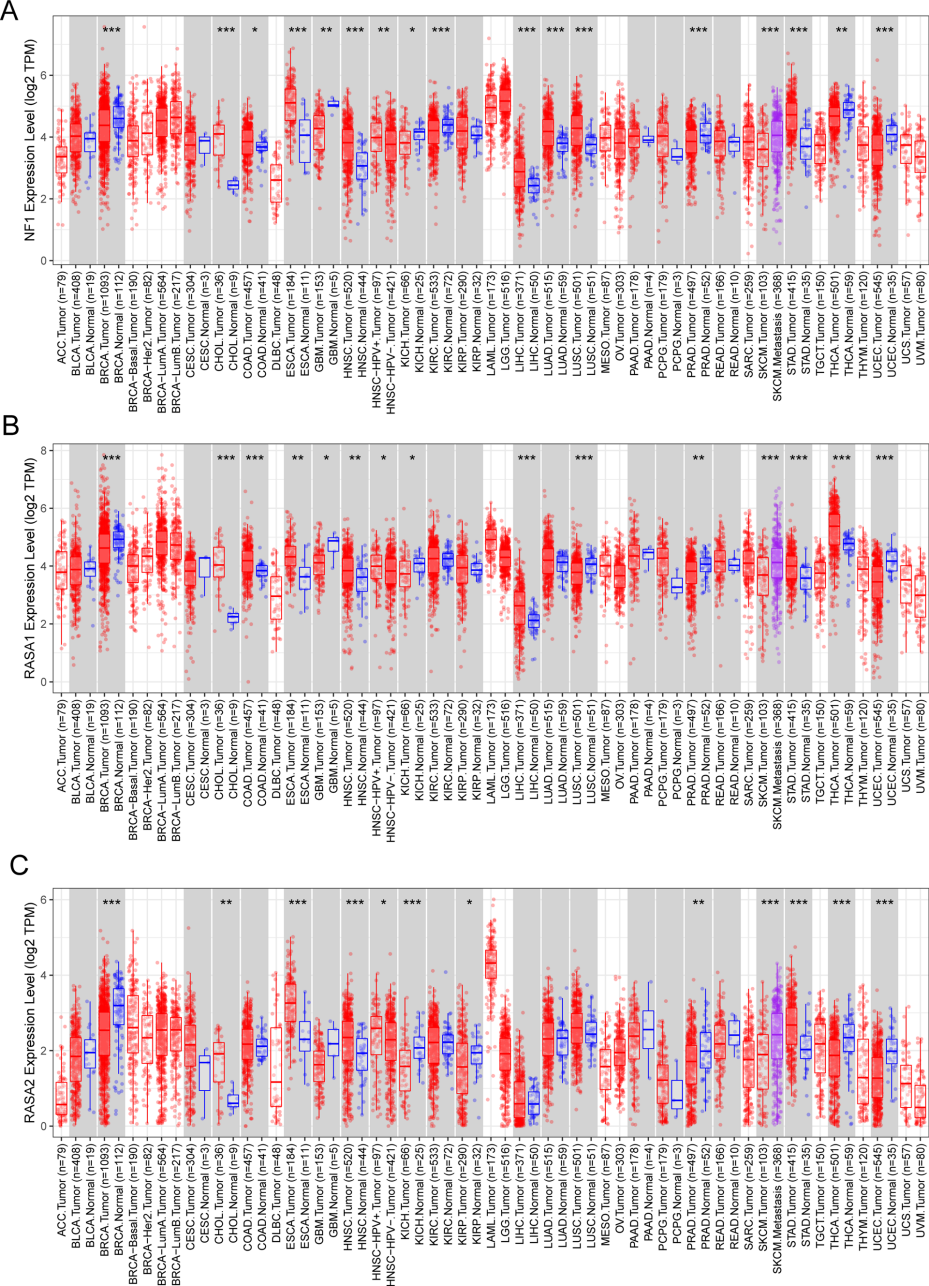
The pan-cancer analysis of (**A**) NF1, (**B**) RASA1 and (**C**) RASA2

**Fig. 4:**
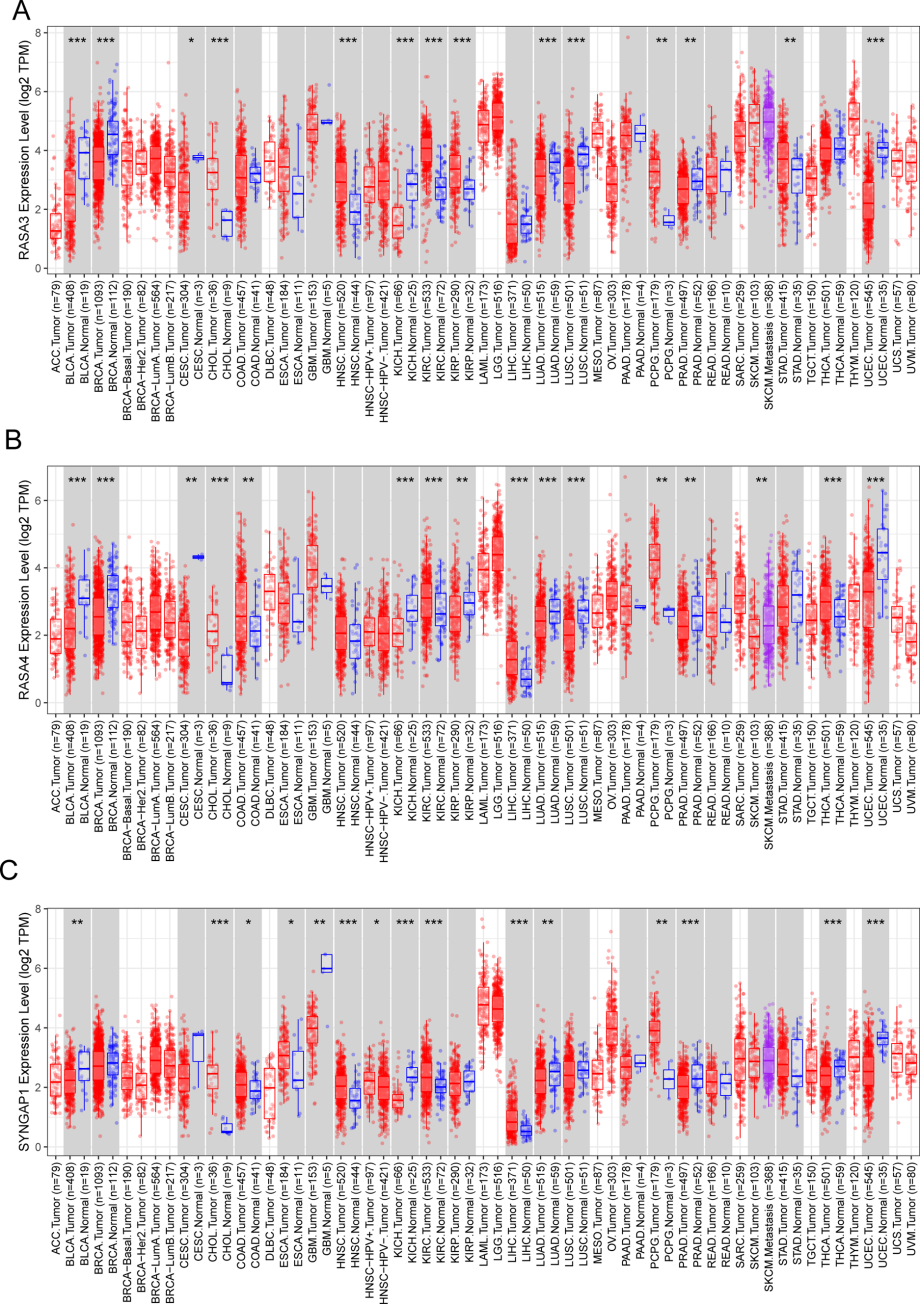
The pan-cancer analysis of (**A**) RASA3, (**B**) RASA4 and (**C**) SYNGAP1

Then, we performed Venn diagram to screen dysregulated Ras GTPase-activating proteins from 5603 DEGs and confirmed 2 dysregulated genes, including RASA4 and SYNGAP1 (Fig. 2A). In addition, the expression pattern of six Ras GTPase-activating proteins was shown, five Ras GTPase-activating proteins including NF1, RASA1, RASA3, RASA4 and SYNGAP1 exhibited a dysregulated level in READ, while only the results of RASA4 and SYNGAP1 were obvious due to their log2 FC > 2 (Fig. 2B). Then, we performed pan-cancer analysis and found that six Ras GTPase-activating proteins exhibited a dysregulated level in many types of tumors(Fig. 3A–C and 4A–C). They showed increased level in tumors, suggesting their anti-oncogenic roles.

### The prognostic value of six Ras GTPase-activating proteins in READ patients

**Fig. 5:**
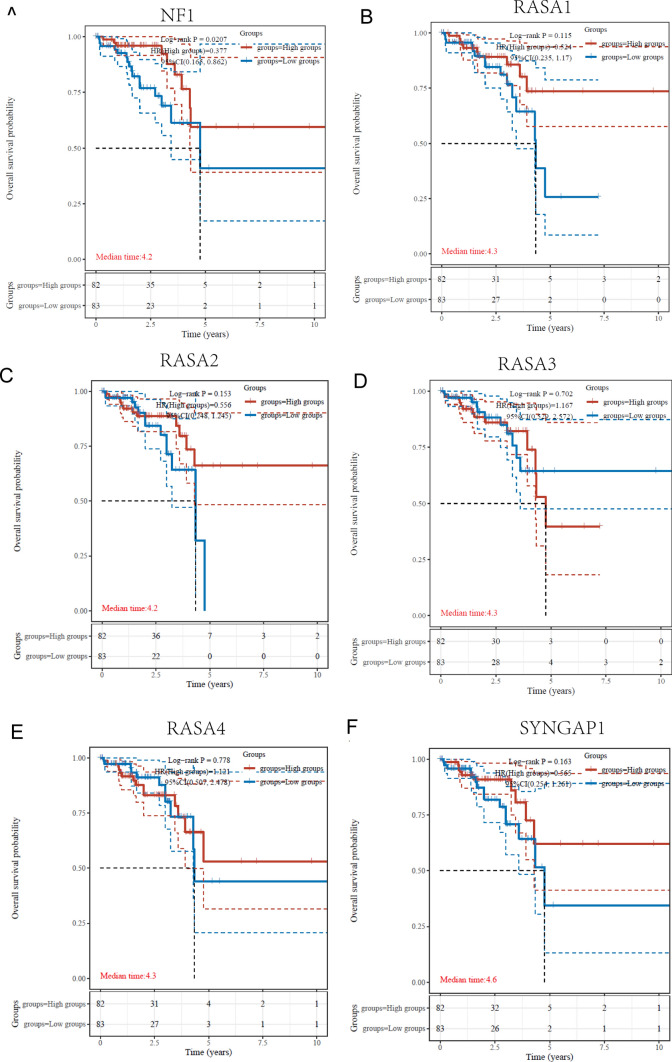
The prognostic value of Ras GTPase-activating proteins in READ patients, including (**A**) NF1, (**B**) RASA1, (**C**) RASA2, (**D**) RASA3, (**E**) RASA4 and (**F**) SYNGAP1

To explore the clinical significance of six Ras GTPase-activating proteins in READ patients, we performed Kaplan–Meier analysis. We observed high expression of NF1 was associated with longer overall survival (Fig. 5A). However, the other five Ras GTPase-activating proteins were not associated with clinical prognosis of READ patients (Fig. 5B–F). Interestingly, patients with high SYNGAP1 expression showed a trend of longer overall survival (Fig. 5F). In summary, NF1 and SYNGAP1 demonstrate certain potential value in the clinical prognosis of READ patients, while the other five Ras GTPase-activating proteins appear to be unrelated to clinical prognosis. These findings provide important clues for further research on the role of these proteins in READ development and treatment, and may offer new insights for personalized therapy and prognosis assessment.

### The analysis of the SNV of Ras GTPase-activating proteins in pan-cancers

**Fig. 6:**
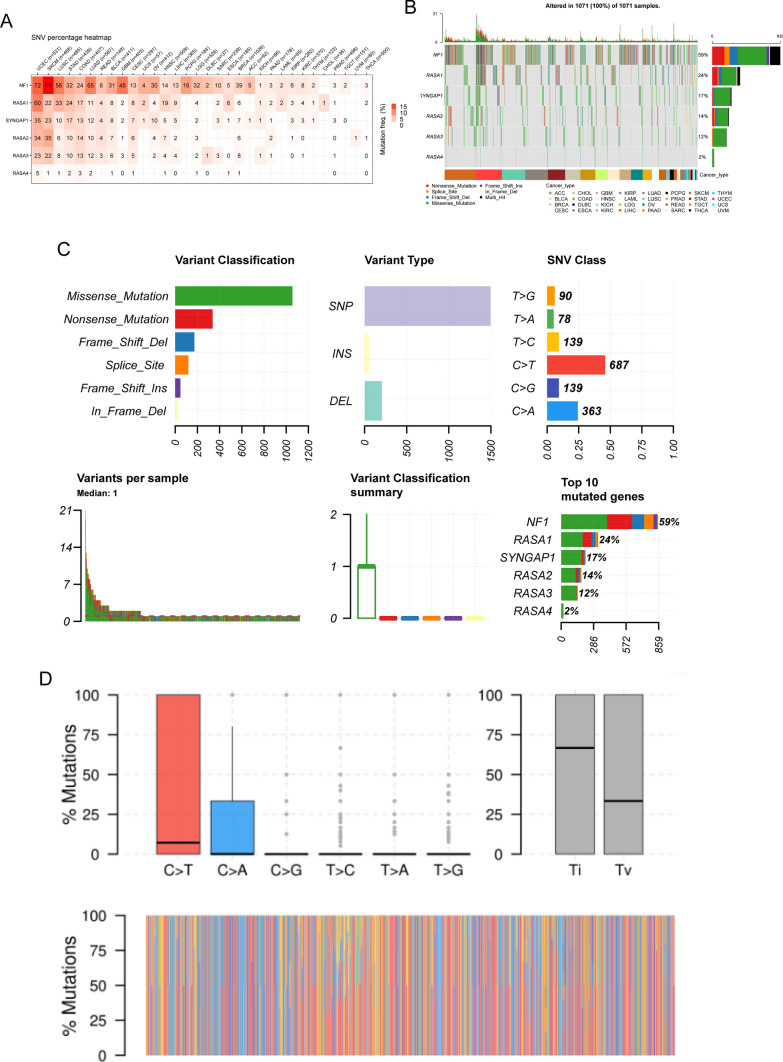
The SNV of Ras GTPase-activating proteins in pan-cancer analysis. **A** The SNV percentage heatmap of Ras GTPase-activating proteins in pan-cancer analysis. **B** The situation of the SNV of Ras GTPase-activating proteins in pan-cancer. **C** and **D** The SNV classes of Ras GTPase-activating proteins in pan-cancer

We investigated the SNV landscape of Ras GTPase-activating proteins in pan-cancer samples because to the strong correlation between gene SNV and cancer development and progression. The results of SNV percentage heatmap revealed that five Ras GTPase-activating proteins had high mutation frequency in UCEC, SKCM, LUSC, STAD, COAD, LUAD and READ (Fig. 6A). According to the analysis, the important 5 mutated genes across cancers were NF1, RASA1, RASA2, RASA3, and SYNGAP1 (Fig. 6B). Furthermore, our findings showed that missense mutations were the most common kind of variant and SNPs were the most common type of mutation in Ras GTPase-activating proteins in pan-cancers (Fig. 6C). Besides, the most abundant SNV class of Ras GTPase-activating proteins was C > A and C > T in pan-cancers (Fig. 6D).

### CNV landscape of Ras GTPase-activating proteins in pan-cancers

**Fig. 7:**
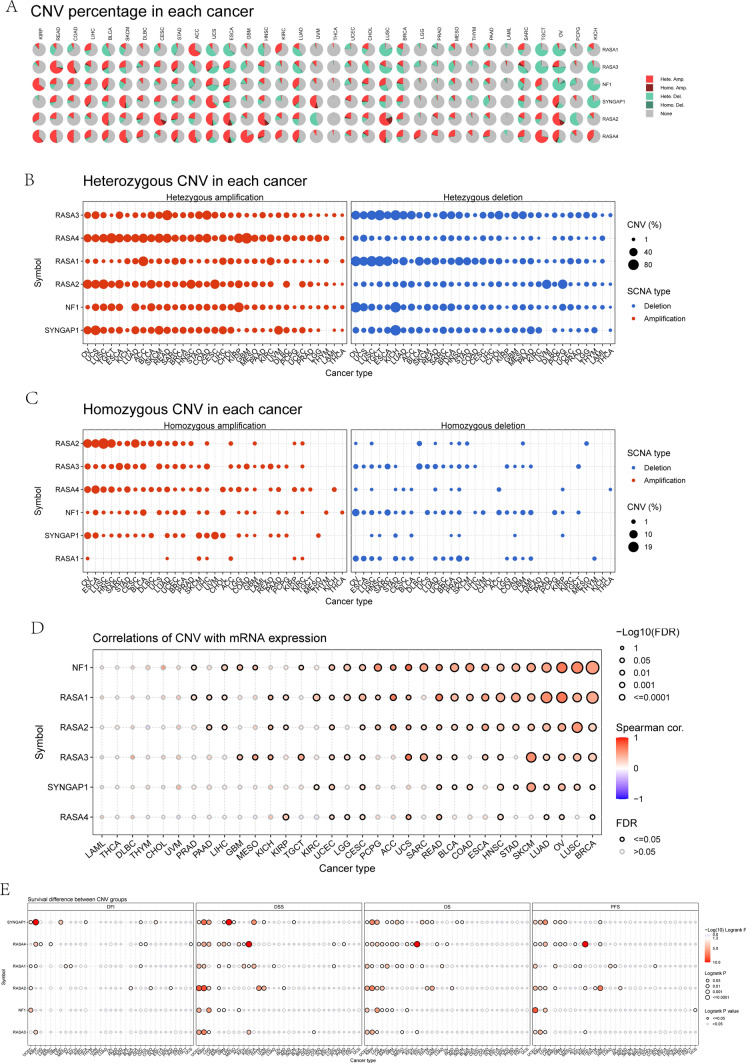
CNV analysis of Ras GTPase-activating proteins in pan-cancers. **A**–**C** The CNV pie plot illustrates the proportion of DEGs that are affected by heterozygous and homozygous copy number variations in pan-cancers. **D** In pan-cancers, the link between CNV and the expression of Ras GTPase-activating proteins. **E** Survival difference between CNV groups of Ras GTPase-activating proteins in pan-cancers

The presence of CNV is a crucial characteristic of the cancer growth process. Next, we set out to investigate the CNV landscape of Ras GTPase-activating proteins in pan-cancers. According to the CNV pie plot, all Ras GTPase-activating proteins had CNV, particularly heterozygous amplification (Fig. 7A–C). We then examined the association between CNV and the expression of Ras GTPase-activating proteins in pan-cancers, and our findings confirmed that the CNV of NF1, RASA1, RASA2, RASA3, and SYNGAP1 was highly correlated with their respective expression in a wide variety of cancers, including BRCA, LUSC, OV, LUAD, SKCM, STAD, HNSC, and others(Fig. 7D). The CNV of several Ras GTPase-activating proteins was shown to affect pan-cancer OS, PFS, DSS, and DFI (Fig. 7E).

### Methylation analyses of Ras GTPase-activating proteins in pan-cancers

**Fig. 8:**
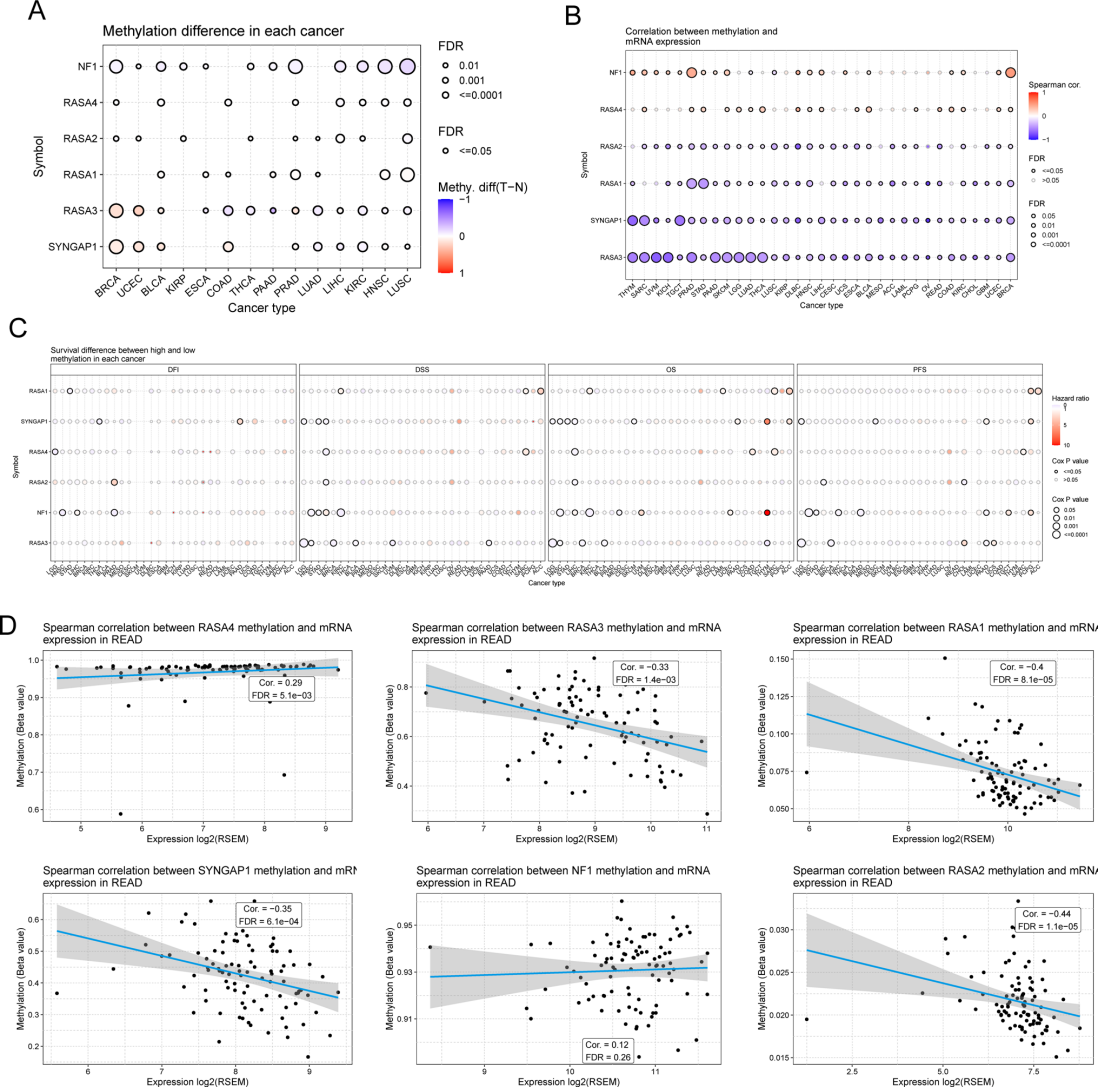
Methylation analyses of Ras GTPase-activating proteins in different cancers. **A** The methylation status of Ras GTPase-activating proteins differs between normal and malignant tissues in several different forms of cancer. **B** Correlation between methylation level and mRNA expression. **C** The effects of methylation on the survival of Ras GTPase-activating proteins in a variety of malignancies. **D** An investigation on the correlation between Ras GTPase-activating proteins and the methylation levels of the READ samples was carried out

Cancer has been linked to DNA methylation for some time now. Therefore, we set out to evaluate how methylation of Ras GTPase-activating proteins varies between various pan malignancies. Firstly, using a bubble plot, researchers were able to see that tumor samples and normal tissues in pan-cancers showed methylation differences for Ras GTPase-activating proteins. (Fig. 8A). Since DNA methylation is known to influence gene expression, we next looked into whether or not methylation of Ras GTPase-activating proteins correlated with their levels. The data revealed that across all TCGA cancer types, DNA methylation of Ras GTPase-activating proteins was inversely linked with their mRNA levels (Fig. 8B). Furthermore, the survival study showed that methylation status of mot Ras GTPase-activating proteins did not significantly affect overall survival or progression-free survival in pan-cancers (Fig. 8C). Importantly, we discovered a negative correlation between DNA methylation and expression for RASA1, RASA2, RASA3, and SYNGAP1 in READ (Fig. 8D).

### *Overexpression of SYNGAP1 distinctly suppressed the proliferation of READ cells *via* Wnt/β-catenin signaling*

**Fig. 9:**
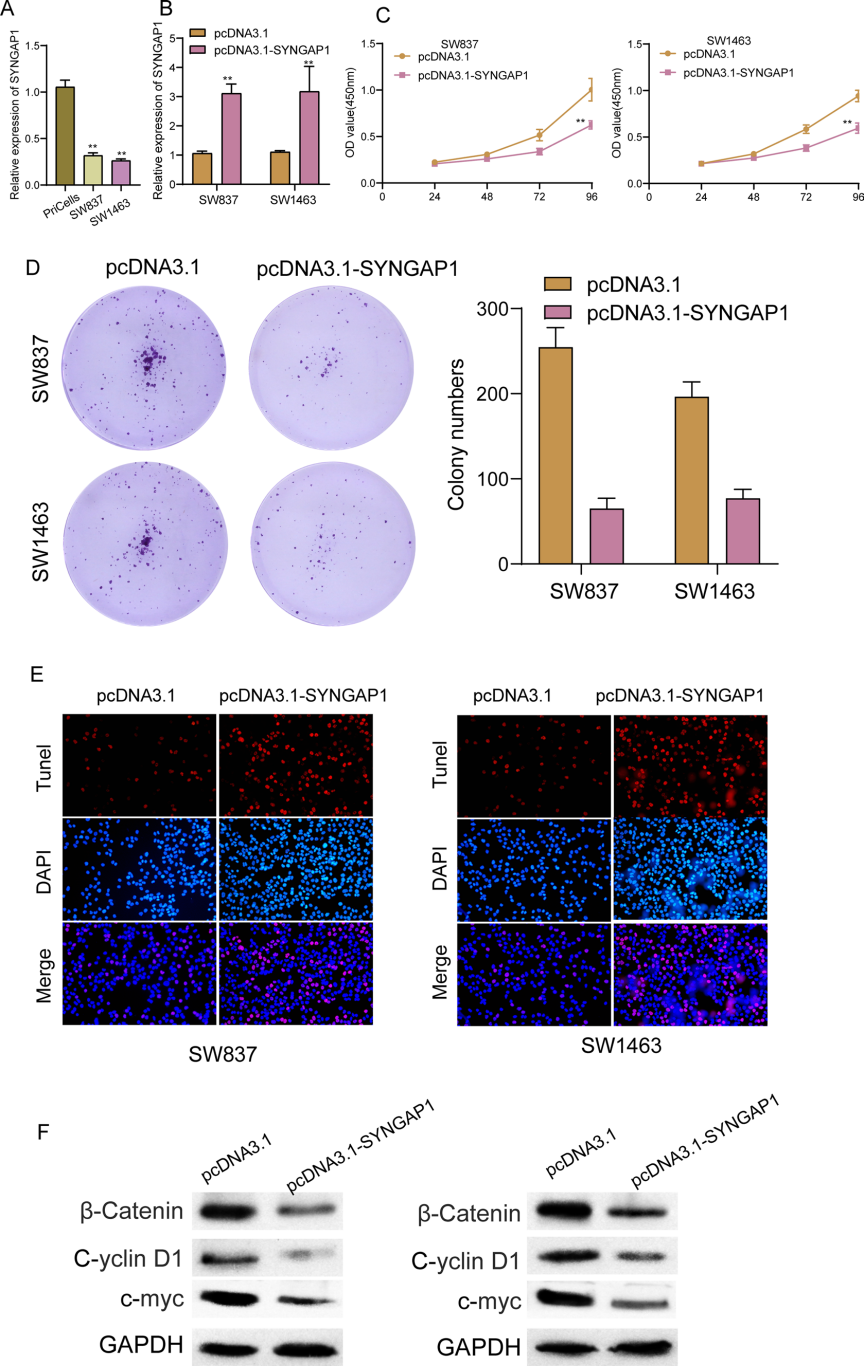
Overexpression of SYNGAP1 distinctly suppressed the proliferation of READ cells via Wnt/β-Catenin signaling. **A** RT-PCR for the expression of SYNGAP1 mRNA in READ cells and normal cells. **B** The overexpression of SYNGAP1 mRNA was confirmed in SW837 and SW1463 was confirmed after the treatment of pcDNA3.1-SYNGAP1. **C** CCK-8 assays. **D** Colony formation assays. **E** Tunel assays. **F** The levels ofβ‐catenin, C-yclin D1 and c-myc after SYNGAP1 overexpression in SW837 and SW1463cells were assessed through western blot

Among six Ras GTPase-activating proteins, our attention focused on SYNGAP1 due to its function was rarely reported in READ. To further confirm the expression of SYNGAP1 in READ, we performed RT-PCR and found that the expression of SYNGAP1 was distinctly decreased in two READ cells (Fig. 9A). In addition, RT-PCR experiments confirmed the transfection efficiency of pcDNA3.1-SYNGAP1 (Fig. 9B). The results of CCK-8 assay and clonogenic assays demonstrated that SYNGAP1 overexpression markedly suppressed the proliferation of SW837 and SW1463 cells (Fig. 9C and D). Moreover, TUNEL assays revealed that SYNGAP1 overexpression significantly increased apoptosis of SW837 and SW1463 cells (Fig. 9E). Furthermore, we examined the expressions of β‐catenin C-yclin D1 and c-myc, which are related to the Wnt/β‐catenin pathway. Our group observed that SYNGAP1 overexpression suppressed the expressions of β‐catenin, C-yclin D1 and c-myc (Fig. 9F).

## Discussion

The prognosis of READ is typically influenced by various factors, with one of the most crucial factors being the cancer’s stage [20, 21]. READ is usually staged using the TNM system, ranging from Stage 0 (earliest) to Stage IV (latest). Early-stage READ (Stages 0 and I) generally has a better prognosis, while late-stage cancer (Stages III and IV) carries a poorer prognosis [22, 23]. Additionally, the location of the tumor can also impact prognosis, with tumors closer to the anus presenting greater treatment challenges and potentially poorer outcomes. The choice of treatment is equally vital for prognosis, as patients who are diagnosed early and receive timely treatment often have a better prognosis. Surgical removal of the tumor is a common treatment approach, but sometimes it may be combined with other treatments such as radiation therapy and chemotherapy [24, 25]. The precise mechanisms underlying the onset and progression of READ remain incompletely elucidated. Nevertheless, gaining a thorough understanding of these mechanisms is of paramount importance for the identification of READ markers and the selection of treatment targets. READ is a heterogeneous disease, with its pathogenesis involving complex interactions between various genetic, environmental, and biological factors. In-depth research into the mechanisms of READ development not only enhances our comprehension of the essence of this disease but also offers crucial insights for the development of more precise treatment strategies and the discovery of novel therapeutic targets.

Ras-GAPs are a class of proteins that play a crucial regulatory role in cellular signal transduction pathways, particularly in signaling related to the activity of Ras proteins [26, 27]. Ras proteins are important GTPases that regulate various biological processes in cells, including cell proliferation, differentiation, and survival. The activity of Ras proteins is regulated by their alternating binding to GTP (guanosine triphosphate) and GDP (guanosine diphosphate) [28, 29]. Ras-GAPs work by promoting the transition of Ras proteins from an active state to an inactive state, thereby inhibiting Ras signal transduction pathways. This is achieved by accelerating the hydrolysis of GTP to GDP in Ras proteins, preventing them from further transmitting signals. Therefore, Ras-GAPs exert negative regulation on cell growth and proliferation [30, 31]. In READ, mutations or abnormal expression of proteins related to the Ras signaling pathway, such as Ras proteins themselves or proteins that interact with them, can lead to the overactivation of this pathway. The abnormal activation of the Ras signaling pathway is associated with the growth and differentiation of cancer cells and may result in uncontrolled proliferation of READ cells [32, 33]. Hence, Ras-GAPs may play a critical regulatory role in the development of READ by influencing the activity of the Ras signaling pathway, ultimately affecting tumor growth and progression. However, the expression pattern and clinical significance of Ras-GAPs remained largely unclear in READ. In this study, we firstly screened DEGs in READ from TCGA datasets and GTEx datasets, and further identified 5603 DEGs. Functional assays revealed that up-regulated genes may be involved in processes related to DNA damage response, immune signal transduction, glycan synthesis, and steroid biosynthesis. On the other hand, down-regulated genes are associated with changes in cyclic nucleotide signaling, muscle contraction, vascular function, and blood pressure regulation. These findings may have significant implications for the potential biological mechanisms at play in your study. For example, the up-regulation of genes related to the p53 signaling pathway suggests the potential activation of cell cycle regulation and DNA damage response, which may be relevant to diseases such as cancer. The down-regulation of genes associated with muscle contraction and vascular function may be linked to cardiovascular or muscle-related disorders. Moreover, we identified two Ras-GAPs(RASA4 and SYNGAP1) which was lowly expressed in READ. In addition, NF1, RASA1 and RASA3 also exhibited a dysregulated level in READ. However, the FC values for these genes were low. The low expression of these Ras-GAPs and their dysregulation in READ may suggest their potential involvement in the development or progression of this cancer. It is known that Ras-GAPs negatively regulate the Ras signaling pathway by promoting the inactivation of Ras proteins, which play roles in various cellular processes, including cell growth and proliferation. Dysregulation of Ras-GAPs could lead to the abnormal activation of Ras signaling, which is associated with uncontrolled cell growth and could contribute to the development of cancer. Moreover, we confirmed that six Ras-GAPs exhibited a dysregulated in many types of tumors. Furthermore, the observation that six Ras-GAPs exhibited dysregulation in various types of tumors suggests a broader significance of these genes in cancer biology. Our findings revealed the significance of Ras-GAPs in cancer development, as their dysregulation can lead to abnormal activation of the Ras signaling pathway, promoting cell proliferation. The observation that six Ras-GAPs exhibited dysregulation in multiple types of tumors suggests their broad relevance in cancer biology, potentially serving as therapeutic targets. Our findings provided new potential strategies and directions for future cancer treatment.

Then, we further explore the prognostic value of six Ras-GAPs in READ patients. We just found that high NF1 expression was associated with longer overall survival. In addition, we also observed a possible clinical trend that high expression of RASA1, RASA2 and SYNGAP1 predicted a favourable prognosis. NF1 is a crucial regulatory protein primarily responsible for promoting the reduction of Ras GTPase protein activity, thus inhibiting cell growth and differentiation by regulating the activity of Ras protein [34, 35]. Ras GTPase is a pivotal protein in cellular signal transduction, capable of activating multiple signaling pathways involved in cell proliferation and growth. When the NF1 gene undergoes mutations, the function of NF1 is impaired, leading to sustained activation of Ras protein, which in turn affects various cellular biological processes [36]. This mutation is the fundamental cause of NF1 disease, resulting in a range of symptoms in NF1 patients, including neurofibromas, skin lesions, skeletal abnormalities, and optic gliomas, among others [37, 38]. We firstly reported the potential of NF1 used as a novel prognostic biomarker for READ patients.

SNV is one of the most common types of genetic variations in the genome and typically involves the replacement, insertion, or deletion of a single nucleotide. SNV is of significant importance in cancer research [39]. Tumors are diseases caused by various genetic and gene mutations, and SNVs are a prevalent type of genetic variation in this context. SNVs can include those that lead to cancer-causing mutations. These mutations may occur in tumor suppressor genes or oncogenes, leading to abnormal cell growth and division, thereby promoting tumor formation [40, 41]. SNV information can be used to develop personalized cancer treatment strategies. By analyzing SNVs in a patient's tumor, doctors can choose more suitable drug treatment options, such as targeted cancer drugs tailored to specific SNV mutations. Based on the study of the SNVs landscape of Ras GTPase-activating proteins in various cancer samples, the following key conclusions can be drawn: In multiple cancers, including endometrial carcinoma, skin melanoma, lung squamous cell carcinoma, stomach adenocarcinoma, colorectal adenocarcinoma, lung adenocarcinoma, and rectal adenocarcinoma, five Ras GTPase-activating proteins exhibited a high mutation frequency. Across different cancers, the five important mutated genes are NF1, RASA1, RASA2, RASA3, and SYNGAP1. Mutations in these genes may play a critical role in tumor development and progression. Furthermore, the mutations in Ras GTPase-activating proteins primarily involve missense mutations and Single Nucleotide Polymorphisms (SNPs), with the most abundant SNV categories being C > A and C > T in various cancers. These findings are expected to enhance our understanding of the mechanisms underlying cancer development and provide valuable insights for personalized treatment and the development of new therapeutic strategies. However, further functional studies and clinical validation are required to confirm the biological and clinical significance of these discoveries.

CNV are a part of genomic polymorphism and are commonly observed among different populations and individuals [42]. Research has shown that CNVs can play a role in the pathogenesis of various diseases, including cancer, autoimmune disorders, and neurological diseases [43, 44]. Therefore, the study of CNVs is of significant importance for understanding genetic diseases, biological processes, and individual differences. Based on the research findings, an in-depth analysis of CNV in Ras GTPase-activating proteins across various cancers was conducted. The study revealed that CNVs are prevalent in different cancers, with heterozygous amplification being the predominant type. Additionally, a close association between CNV and the expression of Ras GTPase-activating proteins was observed, especially in various cancer types such as breast cancer, lung squamous cell carcinoma, and ovarian cancer. Survival analysis results indicated that CNVs in certain Ras GTPase-activating proteins significantly affect the survival periods of patients in different cancers, particularly in endometrial carcinoma, interstitial nephritis of renal papillary necrosis, and low-grade glioma. These findings underscore the significance of CNVs in cancer, providing valuable insights for a deeper understanding of cancer pathogenesis, the development of more effective treatment strategies, and individualized patient prognosis assessments.

Methylation is an essential epigenetic modification process involving the addition of methyl groups (consisting of one carbon atom and three hydrogen atoms) to the cytosine residues within DNA molecules, especially at CpG sites, which are specific DNA sequences where a cytosine is followed by a guanine [45, 46]. The binding of transcription factors and other proteins to DNA is inhibited by this change, leading to gene silence. Methylation is closely associated with cancer. During cancer development, there are often abnormal changes in DNA methylation levels. These changes can involve either an increase or decrease in DNA methylation levels, disrupting normal gene expression patterns. In particular, both DNA hypomethylation and hypermethylation can lead to the abnormal expression of key genes, thereby promoting the development of cancer. Moreover, specific genes or gene regions frequently exhibit abnormal methylation in certain cancer types, making such methylation patterns potential markers and diagnostic indicators for cancer [47, 48]. In this study, we found that RASA3 and SYNGAP1 exhibit differential DNA methylation between tumor samples and normal tissues, indicating the variability of DNA methylation across various cancer types. Furthermore, DNA methylation shows a negative correlation with the expression of these proteins, implying that elevated DNA methylation levels are often associated with repressive gene expression. However, in survival analysis, it was observed that the DNA methylation of most Ras GTPase-activating proteins appears to have no significant impact on patients' overall survival and progression-free survival. Nonetheless, in specific cases, such as in colorectal adenocarcinoma (READ), the DNA methylation of certain proteins may hold unique biological significance. These research findings underscore the heterogeneity of DNA methylation in multiple cancer types and its relationship with gene expression, providing valuable insights for a deeper understanding of the mechanisms underlying tumor development, albeit with varying effects on patient survival across different cancer types.

Given that the expression and function of SYNGAP1 in tumors remained largely unclear, we further explored its function in READ progression. The results of RT-PCR showed that expression of SYNGAP1 was distinctly increased in READ cells, which was consistent with the results from TCGA datasets. Moreover, we confirmed that overexpression of SYNGAP1 distinctly suppressed the proliferation of READ cells, suggesting its anti-oncogenic roles in READ progression. To further studied the potential mechanisms involved in SYNGAP1 on READ progression, our attention focused on Wnt/β-Catenin Signaling. The Wnt/β-Catenin Signaling is a critical cellular signaling pathway that plays a vital role in embryonic development, tissue regeneration, and adult physiological processes [49]. This pathway is named after its two core components, Wnt proteins and β-Catenin proteins. It regulates various biological processes by controlling gene expression and cell fate. In certain circumstances, the abnormal activation of the Wnt/β-Catenin signaling pathway can promote the development of cancer [50–52]. This often involves the excessive accumulation of β-Catenin protein and its increased activity within the cell nucleus. This leads to the overexpression of oncogenes, thereby promoting cell proliferation, growth, and the formation of tumors. In many types of cancer, key components of the Wnt/β-Catenin signaling pathway frequently undergo mutations. These mutations may affect Wnt proteins, receptors, extracellular regulatory factors, or intracellular signaling molecules. These mutations can result in the activation of the signaling pathway, driving tumor growth [53, 54]. In this study, we found that SYNGAP1 overexpression obviously suppressed the protein levels of Wnt/β-Catenin signaling key factors including β‐catenin, C-yclin D1 and c-myc in both SW837 and SW1463 cells, suggesting SYNGAP1 may influence READ progression via regulating Wnt/β-Catenin signaling.

Several potential limitations that may exist in this study. Firstly, we analyzed RNA sequencing data from 165 patients with READ and 789 normal tissue samples. While this is a considerable dataset, a larger sample size could provide more reliable conclusions and enhance generalizability. Secondly, cancer progression is influenced by a multitude of genetic, epigenetic, and environmental factors. While the study focused on SYNGAP1 and its role in READ via the Wnt/β-Catenin signaling pathway, other molecular mechanisms and genetic alterations may also contribute to READ development and progression. Thirdly, we evaluated the short-term effects of SYNGAP1 overexpression on READ cell proliferation and apoptosis. However, long-term effects and potential treatment resistance were not thoroughly investigated. In the future, we will design more sophisticated experiments to study the functions of SYNGAP1.

## Conclusion

We performed integrative analysis and showed the expressing pattern of Ras-GAPs and their clinical values in READ patients. Importantly, we identified SYNGAP1 as a novel tumor suppressor in READ progression via regulating Wnt/β-Catenin signaling. The discovery of SYNGAP1 as a tumor suppressor not only advances our understanding of READ pathogenesis but also reveals potential therapeutic targets. By elucidating the mechanistic link between SYNGAP1 and the Wnt/β-Catenin pathway, our research provides a novel avenue for developing targeted therapies against this signaling axis, thus opening up new avenues for READ treatment. Additionally, our study underscores the importance of personalized medicine in cancer management. The identification of SYNGAP1 as a key player in READ progression highlights the potential utility of molecular profiling and targeted therapies tailored to individual patients' genetic and molecular characteristics. Future research should further explore the mechanistic basis of SYNGAP1-mediated tumor suppression and investigate its therapeutic significance in preclinical and clinical settings. Our finding has the potential to open up new avenues for therapeutic development and personalized treatment strategies for individuals with READ.

### Supplementary Information

Additional file1 (PDF 2929 KB)

## Data Availability

The datasets generated during and/or analyzed during the current study are available from the corresponding author upon reasonable request.
