# Supplementary figures and images for: Overexpression of SYNGAP1 suppresses the proliferation of rectal adenocarcinoma via Wnt/β-Catenin signaling pathway
Source: Discov Oncol. 2024 Apr 29;15:135. doi: 10.1007/s12672-024-00997-z (PMC11056356; doi:10.1007/s12672-024-00997-z)

Supplementary Material

GAPDH

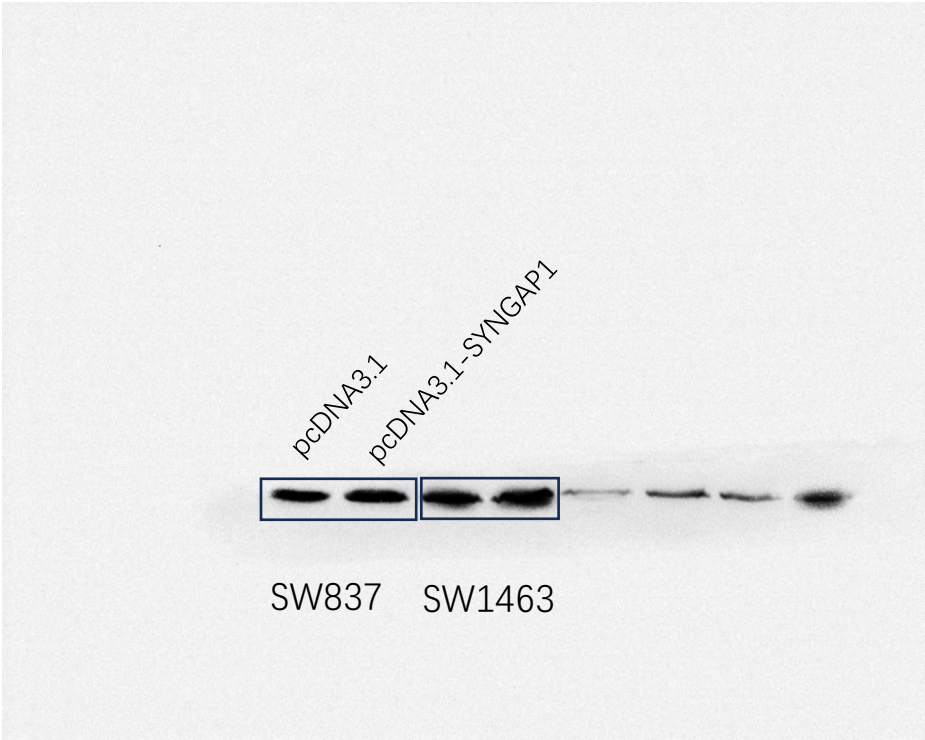

B-Catenin

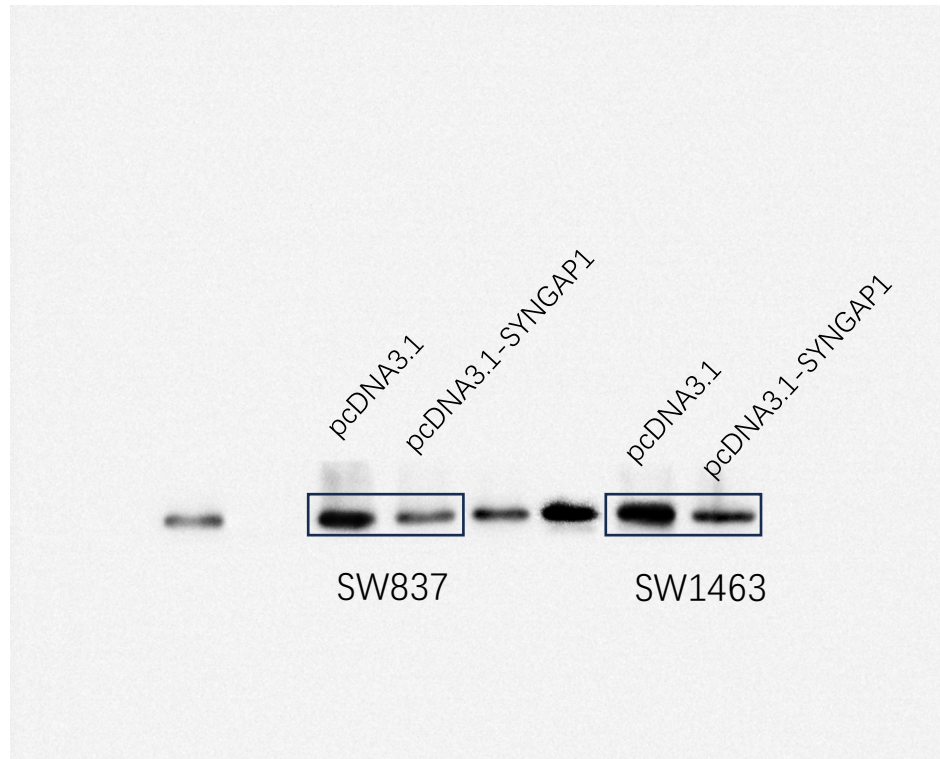

c-myc

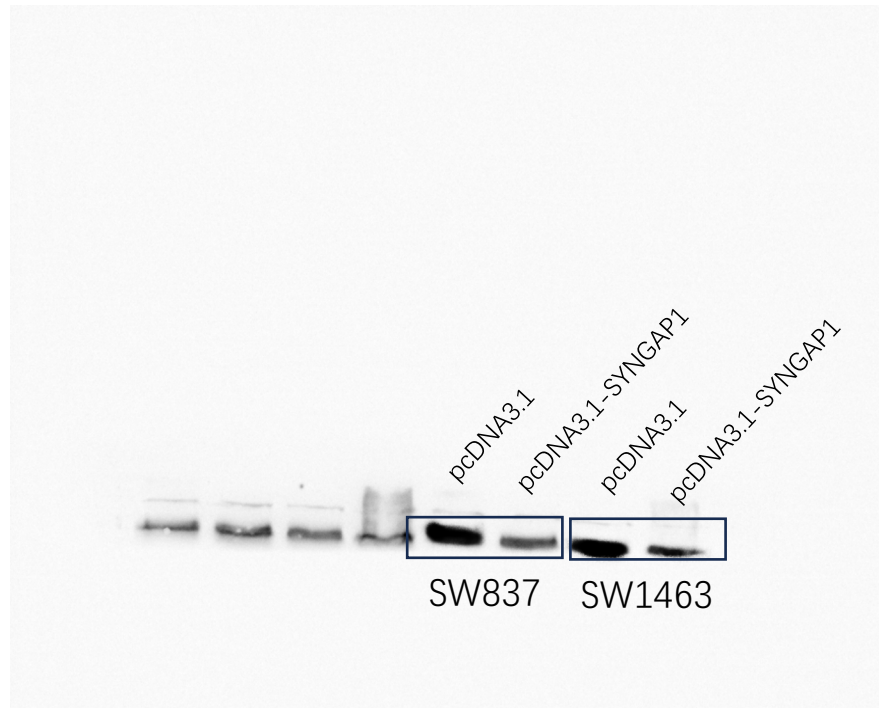

Cycin-d1

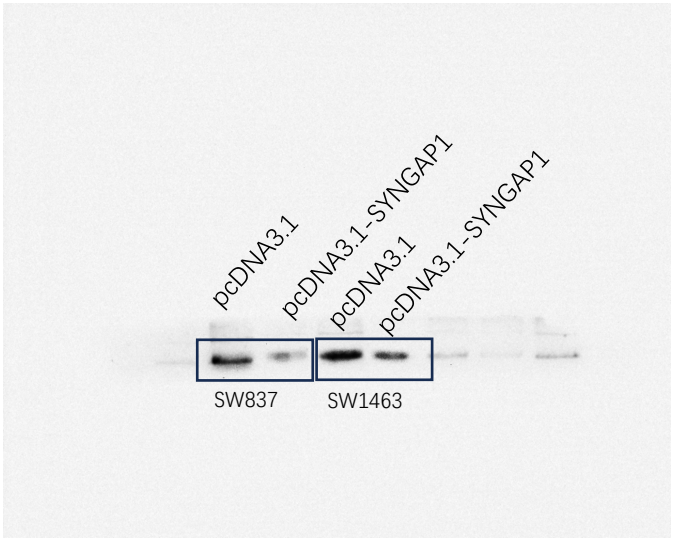

Supplement: Supplementary file 1 — Additional file1 (PDF 2929 KB) [file 12672_2024_997_MOESM1_ESM.pdf]
